# Dr. Aloysius O. J. Kelly

**Published:** 1911-04

**Authors:** 


Dr. Aloysius O. J. Kelly, of Philadelphia died at his home February 23, 1911, aged 41 years. Dr. Kelly was assistant professor of medicine in the University of Pennsylvania, assistant physician to the University Hospital, and professor of pathology in the Woman's College of Philadelphia. He was editor of the American Journal of the Medical Sciences, and the author of "The Practice of Medicine" recently published.

